# Supplementary material for: Canagliflozin Inhibited the Activity of Hemolysin and Reduced the Inflammatory Response Caused by Streptococcus suis
Source: Int J Mol Sci. 2023 Aug 22;24(17):13074. doi: 10.3390/ijms241713074 (PMC10487456; doi:10.3390/ijms241713074)
Supplement: Supplementary file 1 [file ijms-24-13074-s001.zip › ijms-2548570-supplementary.pdf]

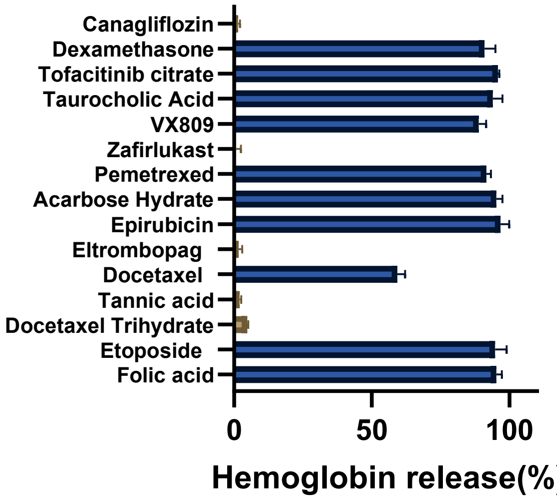

Figure S1. A screening was performed to identify compounds with >50% inhibition of SLY protein hemolysis at a concentration of 64 μg/mL

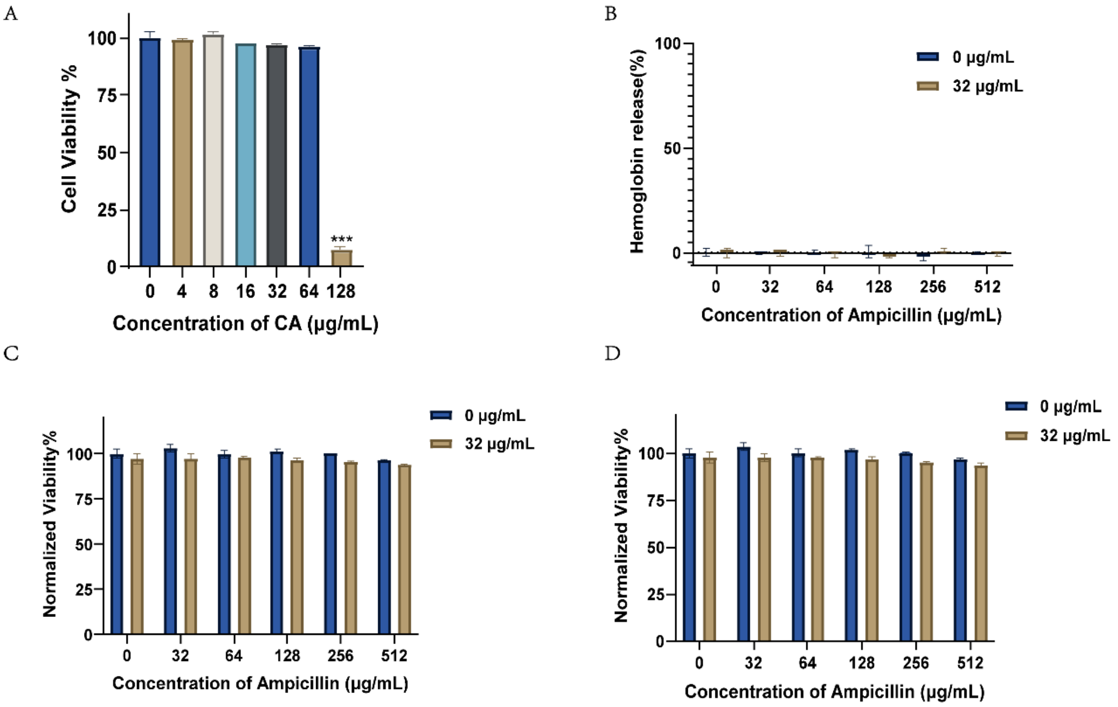

Figure S2. Safety assessment of Canagliflozin. (A)Cytotoxicity of canagliflozin for RAW264.7 *** $p < 0.001$ vs. 0 μg/mL; (B)Hemolytic activity of ampicillin to the RBCs in the absence or presence of canagliflozin; To evaluate Ampicillin cytotoxicity in Vero (C) and RAW264.7 cells(D) in presence of canagliflozin.
